# Population-based cohort study of outpatients with pneumonia: rationale, design and baseline characteristics

**DOI:** 10.1186/1471-2334-12-135

**Published:** 2012-06-18

**Authors:** Dean T Eurich, Sumit R Majumdar, Thomas J Marrie

**Affiliations:** 1 Department of Public Health Sciences, School of Public Health, University of Alberta, Edmonton, Alberta, Canada; 2 Department of Medicine, Faculty of Medicine and Dentistry, University of Alberta, Edmonton, Alberta, Canada; 3 Department of Medicine, Faculty of Medicine, Dalhousie University, Halifax, Nova Scotia, Canada

## Abstract

**Background:**

The vast majority of research in the area of community-acquired pneumonia (CAP) has been based on patients admitted to hospital. And yet, the majority of patients with CAP are treated on an ambulatory basis as outpatients, either by primary care physicians or in Emergency Departments. Few studies have been conducted in outpatients with pneumonia, and there is a paucity of data on short and long term morbidity or mortality and associated clinical correlates in this group of patients.

**Methods:**

From 2000–2002, all CAP patients presenting to 7 Emergency Departments in Edmonton, Alberta, Canada were prospectively enrolled in a population-based registry. Clinical data, including pneumonia severity index (PSI) were collected at time of presentation. Patients discharged to the community were then followed for up to 5 years through linkage to the provincial administrative databases. The current report provides the rationale and design for the cohort, as well as describes baseline characteristics and 30-day morbidity and mortality.

**Results:**

The total sample included 3874 patients. After excluding patients who were hospitalized, died or returned to the Emergency Department the same day they were initially discharged (n = 451; 12 %), and patients who could not be linked to provincial administrative databases (n = 237; 6 %), the final cohort included 3186 patients treated according to a validated clinical management pathway and discharged back to the community. Mean age was 51 (SD = 20) years, 53 % male; 4 % resided in a nursing home, 95 % were independently mobile, and 88 % had mild (PSI class I-III) pneumonia. Within 30-days, return to Emergency Department was common (25 %) as was hospitalization (8 %) and 1 % of patients had died.

**Conclusions:**

To our knowledge, this represents the largest clinically-detailed outpatient CAP cohort assembled to date and will add to our understanding of the determinants and outcomes in this under-researched patient population. The rich clinical data along with the long term health care utilization and mortality will allow for the identification of novel prognostic indicators. Given how under studied this population is, the findings should aid clinicians in the routine care of their outpatients with pneumonia and help define the next generation of research questions.

## Background

### Rationale

Community-acquired pneumonia affects millions of people and results in 1.2 million hospital admissions in the United States each year [1]. In Canada, pneumonia accounts for 1 million physician visits and is a major leading cause of death with ~8,000 deaths per year, most of which occur in the elderly population [2]. An estimated $40 billion is expended annually on pneumonia in the US, including both direct and indirect costs [1]. Over half of patients with community acquired pneumonia (CAP) are treated as outpatients [3] with 10 % subsequently hospitalized [4]. Understanding the prognosis for outpatients with CAP is crucial for optimizing their care [5]. Yet, few studies have evaluated outcomes in CAP outpatients with the majority focusing on short-term mortality and site-of-care decisions. For example, the PORT study investigators evaluated 944 highly selected outpatients and reported a 30-day mortality rate of 0.6 % [6]. More recently, several smaller studies (range of 906 to 1881 patients) have estimated 30-day mortality rates ranging from 0.1 % to 2.5 % [7-10], although a recent large administrative claims study suggest 30-day mortality rates over 4 % among elderly patients [11]. Similarly, few studies have assessed short-term morbidity (e.g., return to Emergency Departments, hospitalization) in CAP outpatients. In the studies conducted to date, admission to hospital following Emergency Department discharge for CAP is between 1.5-8.5 % within 30-days [6,8,10,12,13]. In the only previous study to examine Emergency Department return visits, 3 % of outpatients returned within 30 days [12].

To date, we believe that inadequate information is available for front-line clinicians managing CAP outpatients. Unlike patients admitted to hospital with CAP, little research has been completed on outpatients. In the few studies conducted to date, none have adequately evaluated prognostic factors associated with improved survival or adverse events in CAP outpatients [5]. Thus, numerous inadequacies exist in the extant literature and much of clinical practice in this area has been extrapolated from studies of inpatients. We believe that a large population based cohort of CAP outpatients is urgently needed to begin studying this important but under-researched condition [5]. Therefore, we have assembled such a cohort. In this report, we describe the rationale, key objectives, design and assembly, and characteristics of more than 3000 outpatients with pneumonia.

The key research objectives include, but are not limited to:

1. Understanding the short and long-term health outcomes for patients with CAP managed in an outpatient setting. Outcomes will include mortality, hospitalizations, ambulatory care visits, and physician visits related to recurrent pneumonia and from all-causes.

2. Verifying the utility of various prognostic factors and risk scores commonly used for inpatients with pneumonia and examining them in the outpatient setting.

3. Identifying novel independent prognostic factors associated with significant short and long-term adverse outcomes in CAP outpatients including the impact of comorbidities (e.g. mental health, cardiovascular) and treatment options (e.g., antibiotics) on recovery from an episode of CAP in the community.

4. Describing risk-adjusted long term health care resource use for CAP outpatients

5. Describing the incidence and correlates of “recurrent” pneumonia in those who are not initially admitted to hospital for treatment

6. Examining characteristics of high risk patients who ought to have been admitted to hospital for management rather than discharged home from Emergency Departments (i.e., the low risk patient with relative hypoxemia who is better managed on an in-patient basis);

7. Exploring the impact of impaired functional status and other novel clinical markers that cannot be derived from administrative databases on both short and long-term outcomes

While previous studies have attempted to address some of these issues, available studies are limited by their retrospective assessments [8-12], reliance on administrative data alone, lack of measures of pneumonia severity, scope (e.g., elderly only) [9,11,14], relatively small sample sizes and selection bias [6,8,10,13], selectivity of outcomes (e.g., treatment failure, CAP only hospitalizations) [8,12], short duration of follow-up [6,10,12], and heterogeneity (hospitalized and outpatients combined) [14-16]. Moreover, none of the previous studies have had a sufficient clinically rich population-based sample size to adequately address these relevant questions. We therefore assembled this cohort to improve our current knowledge for outpatients with CAP and help frame future research.

## Methods

### Setting

Between 2000 and 2002 all outpatients with CAP presenting to all 7 Emergency Departments serving Edmonton, Alberta, Canada were enrolled in a population-based clinical registry and treated according to a previously validated clinical management pathway for CAP, and discharged back to the community [17]. Emergency Departments included two tertiary care hospitals, two hospitals that provided secondary and some tertiary care, two smaller community hospitals, and one freestanding urgent-care clinic. The Edmonton health region has a catchment of more than 1 million patients with universal healthcare coverage managed by more than 1000 primary care physicians and has an annual budget of about 2 billion dollars.

### Selection of participants

All patients aged ≥17 years presenting with CAP [defined as two or more of cough, pleurisy; shortness of breath; temperature >38 °C; crackles, or bronchial breathing on auscultation] plus radiographic evidence of pneumonia as interpreted by treating physicians admitted to the Emergency Department and discharge back into the community were enrolled. Patients admitted to hospital or directly to the ICU from the Emergency Department, representing approximately 55 % of our overall cohort, are not included in our outpatient registry [18-20]. All radiographs were subsequently examined by board certified radiologists post-discharge to confirm or refute the presence of opacities consistent with pneumonia. Our registry only excluded patients with tuberculosis, cystic fibrosis, immunocompromised status, hospitalization within the previous 10–14 days, or who were pregnant or nursing. We also excluded patients who were admitted to hospital, died, or returned to the emergency department within the same day to exclude patient transfers, duplicate encounters or hospital admissions or deaths directly related to the initial emergency department encounter. The work was part of a population-wide quality improvement venture and as such, the need for written informed consent was waived. The study was approved by the Ethics Board Panel B of the University of Alberta (Pro00004999).

### Measurements

Research nurses prospectively collected data including sociodemographic (e.g., age, sex, place of residence), clinical (e.g., comorbidities, medications used in the week prior to presentation, functional status, immunization history, smoking status), and laboratory data (Table 1) as well as undertaking a short routine telephone follow-up 1-week post discharge. For most of the information collected, data were classified as abnormal vs. not (as opposed to specific values) according to thresholds specified within the PSI or by community reference values. Furthermore, data collection was necessarily (and by design) not so exhaustive as for inpatients [19,21]. The well-validated pneumonia severity index [PSI] was calculated on all patients at time of presentation [16,22]. For patients with a PSI score >90, or if requested by emergency department physician, an inpatient physician was consulted for admission to hospital.

**Table 1:** Data elements

**1. At time of CAP episode**	**2. Discharge Data**	**3. Follow-up -Data**
**Personal identifier**	**Personal identifier**	**Personal identifier**
personal health number	personal health number	personal health number
**Comorbid illness**		**Comorbid illness**
asthma		asthma
COPD		COPD
heart disease		heart disease
diabetes		diabetes
cancer		cancer
chronic renal failure		chronic renal failure
dementia		dementia
seizures		seizures
stroke		stroke
psychiatric disorder		psychiatric disorder
HIV positive		HIV positive
**Pneumonia Severity Index**		
**Concomitant medications**		**Concomitant medications**
indications		indications
type		type
dose		dose
		duration
**Independent Variables**	**Disposition**	**Primary Outcomes**
hypoxemia, functional status, smoking status, laboratory and diagnostic imaging: (hematology, biochemistry, arterial blood gas, radiographs) Microbiology	discharge disposition discharge destination	Inpatient encounters; admission and discharge dates, diagnostic and procedure codes (ICD-9/10) Physician claims; date and location of service, diagnostic Code (ICD-9/10), provider specialty ambulatory care; date, location of service, Diagnostic and procedure codes (ICD-9/10) Medication Use (> = 65 years); formulary service, prescription date, quantity

### Linkage of clinical registry to administrative databases

For patients discharged, short and longer-term health care resource use and mortality were determined through linkage to various provincial administrative datasets. In Alberta, universal health coverage is provided for the approximately 3.3 million residents. Beneficiaries registered with the Alberta Health Care Insurance Plan receive a unique lifetime personal health number (PHN) that can be linked to various databases. In addition to health services data (hospitalizations, emergency department visits, physician visits), the Ministry of Health maintains and continually updates demographic data on all registered residents including vital statistics through Alberta Vital Statistics death data. A physician or a coroner records cause of death on the death certificate and an algorithm is applied to determine the underlying cause of death according to the World Health Organization on a weekly basis.

**Figure 1:**
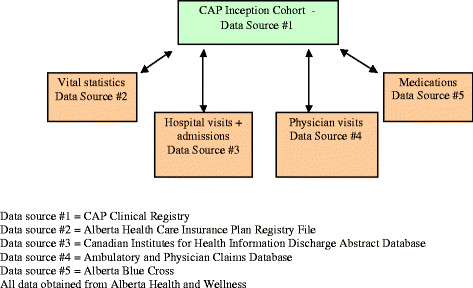
**Data sources.** Data source #1 = CAP Clinical Registry. Data source #2 = Alberta Health Care Insurance Plan Registry File. Data source #3 = Canadian Institutes for Health Information Discharge Abstract Database. Data source #4 = Ambulatory and Physician Claims Database. Data source #5 = Alberta Blue Cross. All data obtained from Alberta Health and Wellness.

Using PHNs, we deterministically linked our population-based cohort of patients to the various provincial health services and vital statistics databases in an anonymous and de-identified manner (Figure 1). As a result, all post discharge individual health service data from the time of study entry to March 31, 2006, were extracted for each patient who was a registered beneficiary in Alberta. These databases are currently being updated to extend our follow-up period to March 31, 2010. All hospitalizations and Emergency Department visits within the province were classified according to International Classification of Diseases, 9^th^ and 10^th^ Revision codes. A major strength of these particular databases compared with other Canadian and US sources is the fact that we can distinguish hospital separations from emergency department visits and from routine physician visits. For those age 65 years and older, we also linked PHNs to the provincial drug databases [23]. This database records information about the drug (based on the American Hospital Formulary System (AHFS)) including class, generic and brand names, strength and dosage, and dates and quantities dispensed, the prescriber, the dispensing pharmacy, and costs. These databases have been used extensively in previous research and are considered by many to be among the highest quality available in Canada [17,18,23]. Data accuracy is routinely validated through provincial and central Canadian agencies.

### Sample size and statistical considerations

Given the large sample size and expected high events rates for the majority of outcomes assessed, we will have more than sufficient power to detect clinically important differences for virtually all clinical correlates of interest. For example, among our sample of over 3000 patients, we will have more than 90 % power (two sided alpha = 0.05) to detect absolute differences in frequencies on the order of 4 %. Alternately, we will have over 95 % power at a two sided alpha = 0.05 to detect a hazards ratio of 1.20 or greater [24]. A 4-5 % absolute difference and 20-25 % relative increases are considered by many (including ourselves) to be clinically important. Moreover, for certain study questions (e.g. rates of lung cancer after pneumonia), we can further increase our sample size by combining the outpatient dataset described here with our previously reported inpatient cohort.

Our analytic plan will follow conventional techniques although we plan to compare and contrast different approaches (e.g., binary and conditional logistic or Poisson regression, competing risk frameworks, recursive partitioning) based on the particular questions asked. Due to the differential length of follow-up for individual patients and some expected losses to follow-up, in general, we will be using a Cox proportional hazards framework throughout (unless otherwise specified) to account for censored patients. Censoring will occur only at the time of death, event(s) of interest, disenrollment from the provincial health insurance program or end of study. Proportional hazards assumptions will be tested using visual inspection, log (−log) plots, and interaction terms with time; if the necessary assumptions are violated, piecewise models will be fitted.

Given the clinical nature of this cohort, it is likely we will not have complete data on all covariates for all patients, although missing data rates in earlier studies of inpatients and ICU patients have been extraordinarily low and for many variables 0-1 % [25,26]. For purposes of analyses, several approaches will be considered for missing data although we cannot address this without more detailed study of the cohort. Potential approaches will include baseline or last value carried forward, missing indicator approaches, and single or multiple imputation [27,28].

## Results

### Preliminary description of the cohort

Overall, 3874 CAP patients were enrolled in our population-based clinical registry. After exclusion of patients admitted to hospital (n = 274, 7 %), died (n = 9, 0.2 %), or who returned to the Emergency Department (n = 168, 4 %) within the same day of discharge, and patients who could not be linked to provincial administrative databases (n = 237; 6 %), the final cohort included 3186 unique patients treated according to a validated clinical management pathway and discharged back to the community. The primary reason for linkage failure was out-of-province health insurance registry (n = 229; 97 %). Few statistically significant or clinically important differences existed between linked and unlinked patients with the exception that linked patients were somewhat more likely to be female (46 % vs. 39 %, p = 0.04) and nursing home residents (5 % vs. 1 %, p = 0.005). Majority of patients were <65 years of age (72 %), 1694 were male (53 %), and 3024 (95 %) were independently mobile. Most patients had a very low predicted risk of 30-day mortality according to PSI score [2811 (88 %) in PSI risk class I – III] (Table 2). Overall, patients with abnormal chest radiographs were very similar compared to those with normal chest radiographs, although the PSI score suggests they may have had slightly more severe pneumonia (Table 3).

**Table 2:** Selected characteristics of 3186 patients admitted to the emergency department for community acquired pneumonia

**Characteristic**	**Baseline N = 3186**
	**Mean ± SD or No. (%)**
Age, yr.	51 ± 20
Age, yr.	
<65	2278 (72 %)
≥65	908 (29 %)
Male	1694 (53 %)
Nursing Home	134 (4 %)
PSI continuous	55 ± 29
PSI Risk Class	
Class I	491 (15 %)
Class II	1858 (58 %)
Class III	462 (15 %)
Class IV	324 (10 %)
Class V	51 (2 %)
Comorbidities	
Diabetes	232 (7 %)
Cardiovascular Disease	523 (16 %)
Malignancy	168 (5 %)
Chronic Kidney Disease	69 (2 %)
COPD	260 (8 %)
Functional Status	
Independent Mobility	3024 (95 %)
Impaired Mobility	162 (5 %)
Influenza vaccination	83 (3 %)
Pneumococcal vaccination	79 (2 %)
Chest Radiograph Confirmation of Pneumonia	1683 (53 %)

PSI = Pneumonia Severity Index; COPD = Chronic Obstructive Pulmonary Disease.

**Table 3:** Selected characteristics according to normal and abnormal chest radiograph

**Characteristic**	**Normal Chest Radiograph (N = 1503)**	**Abnormal Chest Radiograph (N = 1683)**	**p-value**
	**Mean ± SD or No. (%)**		
Death within 30 days	8 (0.5 %)	27 (2 %)	0.004
Hospitalization within 30 days	108 (7 %)	141 (8 %)	0.21
ER revisit within 30 days	385 (26 %)	409 (24 %)	0.39
Age, yr.	52 ± 20	51 ± 20	0.04
Age, yr.			
<65	1049 (70 %)	1229 (73 %)	0.04
≥65	454 (30 %)	454 (27 %)	
Male	801 (53 %)	893 (53 %)	0.90
Nursing Home	60 (4 %)	74 (4 %)	0.57
PSI continuous	54 ± 28	55 ± 30	0.14
PSI Risk Class	251 (17 %)	240 (14 %)	0.04
Class I	862 (57 %)	996 (59 %)	
Class II	222 (14 %)	240 (14 %)	
Class III	153 (10 %)	171 (10 %)	
Class IV	15 (1 %)	36 (2 %)	
Class V			
Comorbidities			
Diabetes	125 (8 %)	107 (6 %)	0.03
Cardiovascular Disease	267 (18 %)	256 (15 %)	0.05
Malignancy	67 (4 %)	101 (6 %)	0.05
Chronic Kidney Disease	29 (2 %)	40 (2 %)	0.39
COPD	125 (8 %)	135 (8 %)	0.76
Functional Status			0.30
Independent Mobility	1433 (95 %)	1591 (95 %)	
Impaired Mobility	70 (5 %)	92 (5 %)	
		
Influenza vaccination	35 (2 %)	48 (3 %)	0.35
Pneumococcal vaccination	32 (2 %)	47 (3 %)	0.23

### Selected outcomes

Overall, 35 (1 %) patients died within 30 days of discharge with the majority in those with chest radiograph confirmation of pneumonia (27 vs 8 with normal radiograph, p = 0.004; Table 3). Readmission to the emergency department or hospitalization within 30 days was high considering the relatively low predicted risk of the cohort. After discharge, 794 (25 %) patients had at least 1 readmission to an Emergency Department for any reason within 30 days. In addition, 249 (8 %) patients were admitted to hospital within 30 days. In terms of duration of follow-up, our cohort represents more than 12 217 person-years with a median follow-up of approximately 1503 days (interquartile range 438).

## Discussion

This prospective population based cohort of CAP patients managed in an outpatient setting is, to our knowledge, the largest clinical cohort assembled and reported to date. Although often considered to be “low risk”, preliminary analyses have shown that short-term morbidity and mortality following discharge is surprisingly elevated. Given the prospective nature of the data, we will be able to fully characterize these patients and identify important and potentially new prognostic factors in this under-studied population. Although well validated “risk scores” have been developed for patients with pneumonia, it is likely that additional important prognostic factors exist that are not fully appreciated [5]. Importantly, given the long-term follow-up data available, we will also be able to fully examine the impact of an episode of pneumonia on long-term sequelae, health care resources use, and prognostic factors associated with improved or adverse outcomes including recurrent episodes of pneumonia.

Despite the strengths of this cohort - large population based clinical cohort of patients managed according to a well-validated clinical pneumonia pathway - there are some limitations. First, despite our large population-based sample, it may not represent all patients with CAP managed in an outpatient setting. For instance, it may not apply to patients evaluated in the primary care setting and discharged home. Second, statistical power for characteristics or events that are rare (<2-3 %) could be an issue. For example, the evaluation of specific therapies on short-term mortality may be problematic due to low short-term mortality rates. Third, although a wealth of information is available within our cohort, reliance on administrative data for longer-term outcomes may not be ideal and we do not have access to continually updated clinical and laboratory measurements. Moreover, it is possible some patients may be lost to follow-up due to out migration from the province and subsequently the administrative datasets. Fourth, we do not have access to microbiology or antibiotic resistance data for this cohort. Fifth, the cohort was generated to be clinically useful and did not rely on a radiologist’s interpretation of opacity consistent with pneumonia on chest radiograph and so there are patients with “normal” radiographs and a clinical diagnosis of pneumonia [29]. While we have access to the reports, we do not have the ability to re-review the actual radiographs. Sixth, although we enrolled a “CAP” cohort, the definition of community-acquired has evolved and some of the patients included in our sample (e.g., recent antibiotics, dialysis patients, nursing home patients in particular) would now be considered to have health-care associated pneumonia. That said, the new definition of health care associated pneumonia is not universally agreed upon and treatment tailored to this entity have increased morbidity and mortality [30]. Last, our clinical registry enrolled patients from 2000–2002 and was limited to Emergency Department patients with universal healthcare coverage managed within a single health region in Canada. Although some advances have been made in the treatment of pneumonia, and patterns of antimicrobial resistance have changed somewhat over the last decade, we strongly believe that almost all aspects of this data will apply to today’s contemporary clinical practice.

## Conclusions

Our cohort of patients will add to our understanding of the impact of an episode of CAP managed in an outpatient setting. Importantly, this data will allow researchers to better understand the determinants of both survival and risk of adverse events in this population. Ultimately, this cohort will help guide future research in the area of pneumonia and will help inform front-line clinicians managing CAP outpatients.

## Abbreviation

CAP, Community Acquired Pneumonia.

## Competing interests

All authors have no association that might pose a conflict of interest (e.g., pharmaceutical stock ownership, consultancy, advisory board membership, relevant patents, or research funding). Sources of support: The clinical registry was funded using an establishment grant from Alberta Heritage Foundation for Medical Research (AHFMR); grants-in-aid from Capital Health; and unrestricted grants from Abbott Canada, Pfizer Canada and Janssen-Ortho Canada (all to TJM). The linked databases with long-term follow-up were funded by Canadian Institutes of Health Research (CIHR) (MOP# 93638 to DTE, SRM, TJM). DTE receives salary support awards from the CIHR and AHFMR; SRM is supported by AHFMR and hold the Faculties of Medicine and Dentistry and Pharmacy and Pharmaceutical Sciences Endowed Chair in Patient Health Management.

## Authors’ contributions

DTE, SRM and TJM all contributed to the study conception and design and acquisition of data. All authors have been involved in drafting the manuscript and revising it critically for important intellectual content; and all authors have given final approval of the version to be published.

## Pre-publication history

The pre-publication history for this paper can be accessed here:

http://www.biomedcentral.com/1471-2334/12/135/prepub
